# Risk factors associated with ticks and *Rickettsia* spp. exposure in wild boars (*Sus scrofa*), hunting dogs, and hunters of Brazil

**DOI:** 10.14202/vetworld.2021.2745-2749

**Published:** 2021-10-25

**Authors:** Louise Bach Kmetiuk, Thiago Fernandes Martins, Renato van Wilpe Bach, Camila Marinelli Martins, Ivan Roque de Barros-Filho, Leandro Cavalcante Lipinski, Giovani Marino Fávero, Andrea Pires dos Santos, Alexander Welker Biondo

**Affiliations:** 1 Graduate College of Cellular and Molecular Biology, Federal University of Paraná (UFPR), Coronel Francisco Heráclito dos Santos Avenue, 100, Curitiba, Paraná State, 81531-970, Brazil; 2 Department of Preventive Veterinary Medicine and Animal Health, School of Veterinary Medicine and Animal Science, University of São Paulo, Orlando de Paiva Street, 87, São Paulo, SP, 05508 270, Brazil; 3 Department of Medicine, State University of Ponta Grossa, Carlos Cavalcante Avenue, 4748, Ponta Grossa, PR, 84030-900, Brazil; 4 Department of Veterinary Medicine, Federal University of Paraná State, Funcionários Street, 1540, Curitiba, Paraná State, 80035-050, Brazil; 5 Department of General Biology, State University of Ponta Grossa, General Carlos Cavalcanti, 4748, Ponta Grossa, Paraná State, 84030-900, Brazil; 6 Department of Comparative Pathobiology, School of Veterinary Medicine, Purdue University, Harrison Street, 725, West Lafayette, Indiana, 47907-2027, USA.

**Keywords:** Brazilian spotted fever, hunting activities, wild boars

## Abstract

**Background and Aim:**

Wild boars have recently been implicated as the maintainers and carriers of *Amblyomma* spp. ticks, which are essential for *Rickettsia* spp. transmission. Consequently, wild boar hunting may increase the risk of tick exposure and subsequent human tick-borne infection and disease. Therefore, this study was conducted to evaluate the risk factors for ticks and *Rickettsia* spp. exposure in wild boars, hunting dogs, and hunters in Brazilian biomes.

**Materials and Methods:**

The statistical relationship of *Rickettsia* spp. antibodies were evaluated using the Chi-square test in 80 wild boars, 170 hunting dogs, and 49 hunters.

**Results:**

The only statistically significant difference in seropositivity found in this study was between male and female wild boars (p=0.034), probably associated with in-park exposure to *Amblyomma brasiliense* infected with *Rickettsia* spp.

**Conclusion:**

The absence of statistical differences in the associated risk factors for hunting dogs and hunters may indicate a random exposure to *Rickettsia* spp.

## Introduction

In recent years, wild boars have been implicated as the maintainers and carriers of *Amblyomma* spp. ticks in Brazil, which are essential for the transmission of *Rickettsia* spp., particularly Brazilian spotted fever (BSF) [1]. Wild boars are classified as exotic invasive species by Brazilian laws, and their hunting has been permitted nationwide, primarily using hunting dogs (*Canis familiaris*) for tracking and hunting [2,3].

Spotted fever has been considered as the most fatal tick-borne disease worldwide, and wild boars may spread infected ticks with *Rickettsia* spp. from their original habitats to other ecosystems, causing human exposure, particularly in specific human activities such as wild boar hunting [1]. Moreover, hunting dogs may elevate the risk of human infection when bringing infected *Amblyomma* spp. ticks back home [4].

Therefore, this study was conducted to evaluate the risk factors for exposure to ticks and *Rickettsia* spp. in wild boars, hunting dogs, and hunters in two different Brazilian biomes.

## Materials and Methods

### Ethical approval

This study was approved by the Ethics Committee of Animal Use (protocol number 059/2017) of the Federal University of Paraná, officially included as part of the annual activities of the City Secretary of Health at Ponta Grossa and approved by National Human Ethics Research Committee (number 97639017.7.0000.0102). In-park trapping and tick collection have been authorized by the Environment Institute of Paraná (authorization number 30/17) and by Chico Mendes Institute of Biology (authorization number 61805–2).

### Study period and location

The present study was conducted from November 2016 to May 2018. The risk of BSF was evaluated on preserved and degraded areas in the Atlantic Forest biome of South Brazil, including the Vila Velha State Park, and degraded areas in the Cerrado biome of Central-West Brazil, as reported previously [1].

### Sampling

This investigation was a descriptive cross-sectional study of risk factors associated with *Rickettsia* spp. and parasitized ticks infesting wild boars, hunting dogs, and hunters [1].

Blood samples of wild boars, hunting dogs, and hunters were collected from October 2016 through May 2018. Blood samples from free-range wild boars in degraded areas were collected after killing them by a firearm according to Brazilian environmental laws. Furthermore, free-range wild boars from the State Park natural areas of Vila Velha were baited, trapped, and killed by a firearm. Blood samples from all wild boars were collected immediately after death by the intracardiac puncture. Blood samples from hunting dogs were collected by jugular puncture as approved by the Ethics Committee of Animal Use at the Federal University of Paraná (protocol 059/2017). Finally, the blood samples of hunters were collected by cephalic puncture as approved by the Ethical Appreciation at Ethics Committee in Human Health at the Brazilian Ministry of Health (protocol 97,639,017.7.0000.0102). All blood samples were collected in tubes without anticoagulants and maintained at room temperature (25°C) until visible clot retraction, after which they were centrifuged at 1500 rpm for 5 min and the resulting serum was stored at −20°C until processing. In addition, ticks were collected from wild boars, hunting dogs, and hunters as approved by the Chico Mendes Institute of Biology.

Serum samples of wild boars, hunting dogs, and hunters were evaluated for the presence of *Rickettsia amblyommatis* strain Ac37, *Rickettsia bellii* strain CL, *Rickettsia parkeri* strain At24, *Rickettsia rickettsii* strain Taiaçu, and *Rickettsia rhipicephali* strain HJ5 by indirect immunofluorescent antibody assay, as described previously [1]. Sera samples were screened at a 1:64 dilution against each of the rickettsial antigens, and in a seropositive reaction, serial dilutions at 2-fold increments were tested up to the endpoint titer. Ticks were randomly collected from wild boars, hunting dogs, and hunters, subjected to DNA extraction, and individually tested by standard polymerase chain reaction for tick mitochondrial 16S rRNA and rickettsial *gltA* gene, as described earlier [1].

The statistical relationship of *Rickettsia* spp. antibodies were evaluated using the Chi-square test in 80 wild boars, 170 hunting dogs, and 49 hunters. For wild boars, the associations tested were between positivity and sex, age, body size, captured in anthropized and/or natural area, and biome of capture; for hunting dogs, the evaluated associations were between positivity and sex, age, biome location, mobility, hunting frequency, hunting experience, and animal hygiene; and for hunters, the associations tested were between positivity and sex, age, household location, biome location, occupation, education, hunting method, hunting frequency, hunting experience, frequency of access to forest areas, observation of capybaras (*Hydrochoerus hydrochaeris*) and opossums (*Didelphis* spp.), number of hunters and family members at the household, hunting dog contact, own a hunter dog, ticks collected, previous tick contact, infestation, control and bites, and activities after hunting.

## Results

A total of 80 wild boars, 170 hunting dogs, and 49 hunters were sampled. Serological analysis for *Rickettsia* spp. showed that 58/80 (72.5%) wild boars, 24/170 (14.1%) hunting dogs, and 5/34 (14.7%) hunters were seroreagent for *Rickettsia* spp., as previously reported [1].

Wild boar exposure to *Rickettsia* spp. was statistically significant in terms of sex, with females being more likely to be positive (p=0.034); however, this was not associated with other risk factors, including age, area of capture, and between free-range and captured wild boars (Table-1). Hunting dog exposure to *Rickettsia* spp. antibodies showed no statistically significant differences, including sex, age, body score, hygiene, vaccination, limited dog mobility, hunting experience in years, and hunting frequency (Table-1).

**Table-1:** Risk factors for seropositivity of anti-*Rickettsia* spp. antibodies in wild boars, hunting dogs, and hunters.

Risk factors of *Rickettsia* spp.	Total	Positive	OR (95% CI)	p-value
	
	Yes/total (%)	Yes/total (%)		
**Wild boar variables**				
Sex				
Female	51/80 (63.7)	41/51 (80.4)	2.89 (1.05-7.96)	0.034
Male	29/80 (36.2)	17/29 (58.6)	(ref)	
<6 months	18/80 (22.5)	13/18 (72.2)	(ref)	
Age				
<6 months	18/80 (22.5)	13/18 (72.2)	(ref)	
>6 months-<1 year old	14/80 (17.5)	10/14 (71.4)	1.04 (0.31-3.48)	0.955
1 year old	48/80 (60.0)	35/48 (72.9)	1.08 (0.29-4.04)	0.913
Free range/captured				
Free range	14/80 (17.5)	11/14 (78.6)	(ref)	
Captured	66/80 (82.5)	47/66 (71.2)	0.67 (0.17-2.69)	0.422
Capture area				
Natural	21/80 (26.2)	21/21 (100)	(ref)	0.390
Agricultural	45/80 (56.2)	26/45 (57.8)	*	
Anthropized	14/80 (17.5)	11/14 (78.6)	*	
Degraded Cerrado	36/80 (45.0)	20/36 (55.6)	*	
Biomes				
Atlantic Forest	21/80 (26.2)	21/21 (100)	(ref)	0.371
Degraded Atlantic Forest	23/80 (28.7)	17/23 (73.9)	*	
Tick collection				
Yes	31/80 (38.7)	26/31 (83.9)	2.76 (0.89-8.49)	0.070
No	49/80 (61.2)	32/49 (65.3)	(ref)	
**Dog variables**				
Age				
<1 year old	27/170 (15.8)	3/27 (11.1)	(ref)	
>1-<8 years old	113/170 (66.5)	15/113 (13.3)	2.00 (0.45-8.94)	0.364
>8 years old	30/170 (17.6)	6/30 (20.0)	1.63 (0.57-4.65)	0.358
Body size				
Small	5/170 (2.9)	2/5 (40.0)	(ref)	
Medium	151/170 (88.8)	20/151 (13.2)	0.25 (0.02-2.58)	0.244
Large	14/170 (8.2)	2/14 (14.3)	1.09 (0.23-5.24)	0.913
Hygiene				
Yes	86/170 (50.6)	9/86 (10.5)	0.54 (0.22-1.31)	0.166
No	84/170 (49.4)	15/84 (17.9)	(ref)	
Vaccination				
Yes	169/170 (99.4)	24/169 (14.2)		0.859
No	1/170 (0.6)	0	(ref)	
Deworming				
Yes	170/170 (100.0)	24/170 (14.1)	-	-
No	0		(ref)	
Dog mobility				
Limited	151/170 (88.8)	23/151 (15.2)	(ref)	
Unlimited	19/170 (11.2)	1/19 (5.3)	0.31 (0.04-2.43)	0.212
Hunting experience				
<1 year	43/170 (25.3)	7/43 (16.3)	(ref)	
>1-<3 years	87/170 (51.2)	12/87 (13.8)	0.73 (0.21-2.53)	0.626
>3 years	40/170 (23.5)	5/40 (12.5)	0.89 (0.29-2.73)	0.842
Hunting frequency (per month)				
Once	29/170 (17.1)	3/29 (10.3)	(ref)	
Twice	63/170 (37.1)	8/63 (12.7)	2.48 (0.34-17.83)	0.368
4 times	69/170 (40.6)	11/69 (15.9)	1.96 (0.35-11.16)	0.446
8 times	9/170 (5.3)	2/9 (22.2)	1.51 (0.28-8.23)	0.636
Tick collection				
Yes	7/170 (4.1)	1/7 (14.3)	1.01 (0.12-8.82)	0.663
No	163/170 (95.8)	23/163 (14.1)	(ref)	
Biome				
Atlantic Forest	147/170 (86.5)	21/147 (14.3)	1.11 (0.30-4.07)	0.586
Cerrado	23/170 (13.5)	3/23 (13.0)	(ref)	
**Hunter variables**				
Sex				
Female	7/49 (14.3)	0/7 (0.0)		0.314
Male	42/49 (85.7)	7/42 (16.6)	(ref)	
Occupation				
Retired and student	8/49 (16.3)	0/8 (0.0)	(ref)	
Private work	32/49 (65.3)	5/32 (15.6)		0.644
Public work	9/49 (18.4)	2/9 (22.2)		0.118
Number of minimum wages				
Up to three	28/49 (57.1)	4/28 (14.3)	(ref)	
Four to eight	16/49 (32.6)	2/16 (12.5)	0.7 (0.1-7.6)	0.744
Up to eight	5/49 (10.2)	1/5 (20.0)	0.6 (0.1-8.0)	0.678
Basic education	5/49 (10.2)	0/5 (0.0)		
School level				
High education	12/49 (24.5)	2/12 (16.7)	(ref)	0.43
Graduate	22/49 (44.9)	2/22 (9.1)		0.151
Postgraduate	10/49 (20.4)	3/10 (30.0)		0.220
Rural household location				
Yes	18/49 (36.7)	3/18 (16.7)	1.3 (0.3-6.8)	0.512
No	31/49 (63.3)	4/31 (12.9)	(ref)	
Registered hunter				
Yes	32/49 (65.3)	6/32 (18.8)	3.7 (0.4-33.5)	0.219
No	17/49 (34.7)	1/17 (5.9)	(ref)	
Forest nearby the household				
Yes	23/49 (46.9)	4/23 (17.4)	1.6 (0.3-8.1)	0.429
No	26/49 (53.1)	3/26 (11.5)	(ref)	
Dog owner				
Yes	5/49 (10.2)	5/38 (13.2)	0.7 (0.1-4.1)	0.500
No	11/49 (22.4)	2/11 (18.2)	(ref)	
Dogs location				
Without dogs	12/49	2/12 (16.7)	(ref)	
Peridomicile	22/49	3/22 (13.6)	0.8 (0.1-5.5)	0.812
Peridomicile and domiciled	2/49	2/14 (14.3)	0.8 (0.1-7.0)	0.867
Domiciled	1/49	0/1		
Contact with dogs of other hunters				
Yes	39/49	6/39 (15.4)	1.6 (0.2-15.4)	0.996
No	10/49 (20.4)	1/10 (10.0)	(ref)	0.559
Presence of capybaras in hunting areas				
Yes	24/49 (49.0)	4/24 (16.7)	1.5 (0.3-7.4)	0.476
No	25/49 (51.0)	3/25 (12.0)	(ref)	
Presence of opossum in hunting areas				
Yes	25/49 (51.0)	3/25 (12.0)	0.7 (0.1-3.4)	0.641
No	24/49 (49.0)	4/24 (16.7)	(ref)	

Hunter exposure to *Rickettsia* spp. antibodies also demonstrated no statistically significant differences, including age, number of years living in rural areas, hunting experience in years, number of people and dogs in hunter household, occupation, income, education level, household location dog owner, peridomicile and dogs maintained indoors, contact with dogs of other hunters, presence of forest areas nearby the household, and presence of capybaras and opossums in hunting areas (Table-1).

The number of *Amblyomma brasiliense*, *Amblyomma sculptum*, *Amblyomma dubitatum*, and larvae of *Amblyomm*a spp. ticks infesting wild boars showed no statistical differences for *Rickettsia* spp. seropositivity when compared between Atlantic Forest with Cerrado biomes and with degraded areas of Atlantic Forest.

Only *Amblyomma aureolatum* ticks were detected in hunting dogs from South Brazil [1], with no statistical significance associated with *Rickettsia* spp. seropositivity. Due to the asymmetric data distribution, the Shapiro–Wilk test (p<0.000001) and the Mann–Whitney U non-parametric test were conducted to confirm the difference in *Rickettsia* spp. seropositivity (20±14; 10, 10-40) and seronegativity (21±14; 10, 10-40) in groups of dogs with no statistically significant outcome (Table-1).

In the present study, 30/49 (61.2%) sampled hunters reported previous tick contact and 23/49 (46.9%) hunters reported tick bites. In addition, 11/30 (36.6%) hunters reported exposure in all seasons, 13/30 (43.3%) hunters reported exposure during summer, 1/30 (3.3%) hunter reported exposure during spring, and 1/30 (3.3%) hunter reported exposure during autumn and winter. A tick check inspection after hunting was reported by 30/49 (59.2%) hunters, and the use of a repellent was reported by only 1/49 (0.2%) hunter. After hunting, 14/49 (28.6%) hunters mentioned taking a shower, and 35/49 (71.4%) hunters reported slaughtering wild boars (Table-1).

## Discussion

In this study, no associated risk factor was statistically significant for wild boars, hunting dogs, or hunters with seropositivity to *Rickettsia* spp., except for wild boar sex, with females being more likely positive (p=0.034). This finding may be due to the high exposure of in-park wild boars, mostly females, because previous research has shown a “forest border effect” risk for dogs and human beings [5].

Hence, the statistically significant differences in seropositivity between 17/29 (58.6%) wild boar males and 41/51 (80.4%) females could be related to the most prevalent wild boar sex and tick species sampled in the natural areas. Not surprisingly, 14/21 (66.6%) wild boar females were slaughtered in-park, all of which were seropositive to *Rickettsia* spp. and had *A. brasiliense* ticks [1].

Although Brazilian hunters may equally hunt male and female wild boars, hunting has been prohibited in state parks, which may serve as a nursery to females [3]. Moreover, male wild boars have demonstrated a higher variation of roaming distance [6], indicating that they are more likely to cross park limits toward surrounding agricultural areas.

The frequency of dog contact with forests has been associated with the occurrence of *Rickettsia* spp. and *Amblyomma* spp. transmission [7]. In the present study, the absence of statistical differences in sex, age, body size, hygiene, vaccination, mobility, hunting experience, and frequency, tick collection, the group size of dogs, and biomes may indicate that hunting dogs are exposed to *Rickettsia* spp., irrespective of the associated risk factors.

Although no study has focused on hunter exposure, people infected with BSF were found to be primarily white males aged between 20 and 64 years from the rural areas of South and Southeast Brazil, visiting natural environments (66.7%), exposed to ticks (72.7%), and contacting capybaras (15.6%), dogs and cats (42.4%), cattle (17.2%), and horses (17.4%) [8]. Therefore, the absence of statistical differences in the present study may indicate that hunters are exposed to *Rickettsia* spp., irrespective of their sex, age, occupation, income, education, household location, owning a dog, contact with hunting dogs, presence of capybaras or opossums, number of years lived in rural areas, hunting experience, and number of people and dogs in the hunter’s household.

Finally, all ticks collected in these areas were negative for the presence of *Rickettsia* spp. as they have rarely infected BSF-non-endemic areas [1]. In Southeast Brazil, high capybara population has been associated with a high tick infestation rate in BSF-endemic areas [9], with wild boar occurrence reportedly overlapping such areas [10].

## Conclusion

The entry of wild boars, hunting dogs, and hunters into tick habitats may lead to bites and consequent infection with *Rickettsia* spp. The only statistically significant association with seropositivity found in this study was the increased risk due to hunting female wild boars, which is probably associated with in-park exposure to *A. brasiliense* and *Rickettsia* spp. Furthermore, the absence of statistical differences in the associated risk factors for hunting dogs and hunters may indicate a random exposure to *Rickettsia* spp.

## Authors’ Contributions

LBK, APS, and AWB: Conceptualization. LBK, TFM, RVWB, CMM, IRB, LCL, GMF, APS, and AWB: Data collection and data analysis. LBK and AWB: Drafted and revised the manuscript. All authors read and approved the final manuscript.
